# Assessment of Outcomes of Treatment With Oral Anticoagulants in Patients With Atrial Fibrillation and Multiple Chronic Conditions: A Comparative Effectiveness Analysis

**DOI:** 10.1001/jamanetworkopen.2018.2870

**Published:** 2018-09-28

**Authors:** Amgad Mentias, Ghanshyam Shantha, Pulkit Chaudhury, Mary S. Vaughan Sarrazin

**Affiliations:** 1 Division of Cardiovascular Medicine, Roy and Lucille J. Carver College of Medicine, University of Iowa Hospitals and Clinics, Iowa City; 2 Department of Internal Medicine, Roy and Lucille J. Carver College of Medicine, University of Iowa, Iowa City; 3 Comprehensive Access and Delivery Research and Evaluation Center (CADRE), Iowa City VA Medical Center, Iowa City

## Abstract

**Importance:**

Comparative effectiveness and safety of oral anticoagulants in patients with atrial fibrillation (AF) and multiple chronic conditions (MCC) are unknown.

**Objective:**

To determine whether there are differences in efficacy and safety of dabigatran, rivaroxaban, and warfarin regarding stroke prevention and bleeding rates, respectively, in elderly patients with AF with MCC.

**Design, Setting, and Participants:**

This retrospective comparative effectiveness analysis included data from the population-based Medicare beneficiaries database, evaluating patients with new AF diagnosed from January 1, 2010, to December 31, 2013, who initiated an oral anticoagulant within 90 days of diagnosis. Patients with CHA_2_DS_2_-VASc scores of 1 to 3, 4 to 5, and 6 or higher; HAS-BLED scores of 0 to 1, 2, and 3 or higher; and Gagne comorbidity scores of 0 to 2, 3 to 4, and 5 or higher were categorized as having low, moderate, or high morbidity, respectively. Within morbidity categories, patients receiving dabigatran, rivaroxaban, or warfarin were matched using a 3-way propensity matching, and the relative hazards of stroke, major hemorrhage (MH), and death were evaluated. Data analysis included follow-up from the date of initial anticoagulant use through December 31, 2013.

**Exposures:**

Rivaroxaban (20 mg once daily), dabigatran (150 mg twice daily), or warfarin therapy.

**Main Outcomes and Measures:**

Ischemic stroke, MH, and death.

**Results:**

The study cohort included 21 979 patients using dabigatran (mean [SD] age, 75.8 [6.4] years; 51.1% female), 23 177 using rivaroxaban (mean [SD] age, 75.8 [6.4] years; 49.9% female), and 101 715 using warfarin (mean [SD] age, 78.5 [7.2] years; 57.3% female). In the propensity-matched cohorts, there were no differences in stroke rates between the 3 oral anticoagulant groups. Dabigatran users had lower hazard of MH compared with warfarin users among patients with low MCC (hazard ratio [HR], 0.62; 95% CI, 0.47-0.83; *P* < .001; for MCC defined as low CHA_2_DS_2_-VASc score), and similar risk in patients with moderate to high MCC. While there was no difference in MH between rivaroxaban and warfarin users, rivaroxaban users had significantly higher MH risk compared with dabigatran users in the medium and high comorbidity groups (HR, 1.24; 95% CI, 1.04-1.48; *P* = .02 and HR, 1.28; 95% CI, 1.05-1.56; *P* = .01, respectively). Dabigatran and rivaroxaban users had lower rates of death compared with warfarin users (HR ranged from 0.52-0.84), across comorbidity levels.

**Conclusions and Relevance:**

Oral anticoagulants are similarly effective in stroke prevention among patients with AF with MCC. However, dabigatran and rivaroxaban use may be associated with lower rates of mortality in patients with MCC.

## Key Points

**Question:**

What are the comparative efficacy and safety of dabigatran and rivaroxaban compared with warfarin in patients with multiple chronic conditions?

**Findings:**

In this comparative effectiveness analysis, stroke rates were similar between the 3 drugs. However, bleeding rates were significantly higher with rivaroxaban compared with the other 2 drugs, while mortality rates were lower with dabigatran and rivaroxaban compared with warfarin.

**Meaning:**

Rivaroxaban and dabigatran are effective in elderly patients with multiple chronic conditions, but dabigatran appears to be associated with lower bleeding rates.

## Teaser

This comparative effectiveness analysis assesses the efficacy and safety of oral anticoagulants dabigatran, rivaroxaban, and warfarin in elderly Medicare beneficiaries with atrial fibrillation (AF) and multiple chronic conditions.

## Introduction

Nonvalvular atrial fibrillation (AF) affects 3 million adults in the United States.^1^ Atrial fibrillation increases stroke risk by 3- to 5-fold.^2,3^ For several decades warfarin was the only chemoprophylaxis that was available for stroke prevention in patients with AF and it reduces stroke risk by nearly 60%.^4^ Recently, direct oral anticoagulants (DOACs) such as dabigatran and rivaroxaban were approved for stroke prevention in AF.^5,6,7^ Randomized clinical trial (RCT) data support the efficacy of these drugs and they are now commonly used in clinical practice.

Although the RCT data supported efficacy of DOACs, extrapolating these results to real-world patients with multiple chronic conditions (MCC) may not be appropriate because such patients were not well represented in the RCTs. In the ROCKET AF trial,^7^ less than 15% of the study participants had a CHADS_2_ score of 5 or higher. In the RE-LY trial, less than one-third of the study participants had a CHADS_2_ score of 3 or higher.^5,6^ Moreover, stroke prevention efficacy and bleeding outcomes of DOACs specific to this high-risk group were not reported as a prespecified analysis in these RCTs,^8^ and observational studies on DOACs have not focused on high-risk patients.^9,10,11^ Despite their increasing use, studies reveal that DOACs are more likely to be initiated in healthier patients with fewer comorbidities.^12,13^ High-risk patients with MCC not only have higher stroke and bleeding risk, but also make up to 50% of newly diagnosed patients with AF.^9^ Therefore, it is critically important to understand the efficacy and safety of DOACs in these patients.

To bridge this literature gap, we used nationally representative Medicare data to study efficacy and safety outcomes of rivaroxaban and dabigatran compared with each other and with warfarin in patients with MCC.

## Methods

The institutional review board of the University of Iowa approved this study and granted a waiver of informed consent owing to the large number of patients and retrospective cohort design. We used the Centers for Medicare & Medicaid Services–linked patient-level administrative data including Beneficiary Summary File Base and Chronic Conditions segments; Inpatient (Part A) and Carrier (Part B) Standard Analytic Files for 2010 through 2013; and Pharmacy Drug Event (Part D) files for 2010 through 2013. We identified 213 705 Medicare beneficiaries who were enrolled in the Centers for Medicare & Medicaid Services Part D prescription drug plan, were newly diagnosed with AF between November 1, 2011, and October 31, 2013, and initiated dabigatran (150 mg) twice daily, rivaroxaban (20 mg) once daily, or warfarin within 90 days after AF diagnosis. New AF was defined as 1 inpatient claim or 2 outpatient claims within 90 days with *International Classification of Diseases, Ninth Revision, Clinical Modification* (*ICD-9-CM*) code 427.31 as primary diagnosis, with no preceding AF diagnosis or anticoagulant use in the prior 12 months.^14,15^ We excluded patients if they were younger than 66 years at the time of diagnosis (to ensure at least 12 months of Medicare eligibility before diagnosis), were enrolled in a Medicare managed care during the observation period, or were not enrolled in a Part D drug prescription plan at the time of AF diagnosis. This study followed the International Society for Pharmacoeconomics and Outcomes Research (ISPOR) reporting guideline for comparative effectiveness research.^16^

### Study Outcomes

The primary outcomes were inpatient admission for acute ischemic stroke or major bleeding as defined by Rothendler et al^17^ and Suh et al^18^ based on the primary *ICD-9-CM* diagnosis on inpatient standard analytical files claims for acute care stays. The secondary outcomes were gastrointestinal hemorrhage (GIH), nongastrointestinal major hemorrhage (including intracranial hemorrhage), acute myocardial infarction (MI) (defined as primary *ICD-9-CM* diagnosis code 410.XX), and death (defined using the date of death of Medicare enrollment files).^14^ All outcomes were specified prior to the initiation of the study.

### Patient Characteristics

Demographics data including patient age, sex, race, and ethnicity were identified from Medicare enrollment data. We identified the presence of specific comorbid diseases defined by Elixhauser et al^19^ using *ICD-9-CM* diagnoses in inpatient and outpatient claims during the 12 months preceding AF diagnosis. Previous cerebrovascular events and bleeding episodes were also identified using published algorithms.^17,18^ We identified additional comorbidities of importance to AF outcomes, including other dysrhythmias (*ICD-9-CM* codes 427.X, excluding 427.3), cardiomyopathy (*ICD-9* codes 425.X), cardiac conduction disorder (eg, bundle branch block; *ICD-9* codes 426.X), and previous implantable cardiac device (eg, pacemaker; *ICD-9* codes V45.0 and V53.3).

To assess the presence of MCC or illness burden in our cohort, we used 3 different scoring systems:

- CHA_2_DS_2_-VASc score: Ranges from 0 to 9 with 1 point assigned for the presence of congestive heart failure, hypertension, age 65 to 74 years, diabetes, vascular disease (including prior MI or peripheral artery disease), and female sex.^20^ Two points are assigned for a history of stroke or aged 75 years or older. Given that all patients in our cohort were 65 years or older, the minimum possible CHA_2_DS_2_-VASc risk score in our cohort was 1. Patients were categorized as having low (score of 1-3), moderate (score of 4-5), or high (score of ≥6) stroke risk.
- HAS-BLED score: Reflects 1-year bleeding risk in patients with AF and has been well validated.^21^ The score ranges from 0 to 9, and assigns 1 point for the presence of hypertension, prior stroke, history or predisposition toward bleeding, aged 65 years or older, concurrent use of alcohol or drugs that increase bleeding risk, liver disease, and renal disease. The score also incorporates an indicator for patients with a history of labile international normalized ratio (INR), which was not relevant for our study since only anticoagulant-naive patients were eligible. Patients were categorized as having low (score of ≤1), moderate (score of 2), or high (score of ≥3) bleeding risk.
- Gagne comorbidity score summarizes comorbidities in terms of mortality risk.^22^ The score incorporates the following comorbid conditions defined by Romano et al^23^ and Elixhauser et al^19^: unexplained weight loss, hemiplegia, alcohol abuse, tumor, renal disease, metastatic cancer, dementia, arrhythmia, pulmonary disease, coagulopathy, complicated diabetes, anemia, electrolyte imbalance, liver disease, peripheral vascular disease, psychosis, pulmonary circulatory disorder, HIV and/or AIDS, and heart failure.^22^ Points are assigned to reflect the risk of death. Patients were categorized as having low, moderate, or high risk of death according to the Gagne comorbidity score (score of 0-2, 3-4, or ≥5, respectively).

### Statistical Analysis

Patients were categorized into rivaroxaban users, dabigatran users, and warfarin users according to the first anticoagulant received after AF diagnosis. In each of these anticoagulant groups, patient characteristics were reported as mean (with standard deviation) or median (interquartile range) for continuous variables, or as number and percentage for categorical variables. We used χ^2^ test or 1-way analysis of variance, as appropriate, to compare demographic variables, comorbid conditions, health care utilization, and medication use, between the 3 anticoagulant groups. Each anticoagulant group was then subdivided into low, moderate, and high morbidity levels, based on alternative MCC definitions. We reported the study outcomes as number of events, percentage, and events per hundred patient-years of follow-up separately by anticoagulant within MCC level.

We then used the 3-way propensity matching method described by Rassen et al^24^ to create groups of patients receiving dabigatran, rivaroxaban, or warfarin that were balanced with respect to patient covariates. The matched cohorts comprised patients with similar observed characteristics overall and roughly equal likelihood of receiving each drug and were therefore plausible candidates for all 3 anticoagulants under study, based on observed patient characteristics. Matching was conducted separately for patients with low, moderate, or high MCC, as defined using the 3 alternative multimorbidity measures (ie, 9 different matching algorithms were conducted). Success of each matching algorithm was evaluated by comparing standardized differences in patient demographic characteristics, comorbidity, medication use, and health services history between each drug in the matched samples. Standardized differences less than 10% (ie, 10% of the standard deviation of the difference) suggest adequate balance.^25^

Within each MCC level, multivariable Cox proportional hazards regression models were generated on the matched samples to further control for possible differences between patients receiving alternative anticoagulants. In these models, the dependent variables were time (in days) from anticoagulant initiation to a given event (eg, admission for stroke or censoring), while candidate independent variables included patient demographic characteristics, comorbid conditions, concurrent medication use, and prior health services use. The eTable in the Supplement provides details regarding the variables included in the adjusted models. Censoring events included end of observation (December 31, 2013), cessation of the initial anticoagulant (defined as the date of the last fill plus days supplied), or death. Variables were selected for inclusion in Cox models based on relationship to the outcome, using a statistical criterion on .05. Models also included indicators for the type of anticoagulant used, which were used to estimate the relative hazard of each outcome in patients taking dabigatran (relative to warfarin), rivaroxaban (relative to warfarin), and dabigatran (relative to rivaroxaban). Data set creation and propensity matching were conducted using SAS. Two-sided *P* < .05 was considered statistically significant.

## Results

Prior to matching, 21 979, 23 177, and 101 715 patients who initiated dabigatran (150 mg), rivaroxaban (20 mg), or warfarin, respectively, met the study eligibility criteria. Dabigatran users had lower hazard of MH compared with warfarin users among patients with low MCC (hazard ratio [HR], 0.62; 95% CI, 0.47-0.83; *P* < .001; for MCC defined as low CHA_2_DS_2_-VASc score), and similar risk in patients with moderate to high MCC. While there was no difference in MH between rivaroxaban and warfarin users, rivaroxaban users had significantly higher MH risk compared with dabigatran users in the medium and high comorbidity groups (HR, 1.24; 95% CI, 1.04-1.48; *P* = .02 and HR, 1.28; 95% CI, 1.05-1.56; *P* = .01, respectively). Dabigatran and rivaroxaban users had lower rates of death compared with warfarin users (HR ranged from 0.52-0.84), across comorbidity levels. Compared with patients who initiated warfarin, patients who initiated dabigatran or rivaroxaban were significantly younger (mean [SD] age, 78.5 [7.2] vs 75.8 [6.4] and 75.8 [6.4] years, respectively), less likely to be female (58 327 [57.3%] vs 11 239 [51.1%] and 11 571 [49.9%], respectively), and less likely to be black or Hispanic (black: 5420 [5.3%] vs 768 [3.5%] and 744 [3.2%]; Hispanic: 4689 [4.6%] vs 824 [3.7%] and 851 [3.7%], respectively). Compared with patients who initiated warfarin, patients who received dabigatran or rivaroxaban were also significantly less likely to have heart failure (37 698 [37.1%] vs 5310 [24.2%] and 5405 [23.3%], respectively), peripheral vascular disease (25 507 [25.1%] vs 4056 [18.5%] and 4335 [18.7%], respectively), diabetes (38 040 [37.4%] vs 7194 [32.7%] and 7468 [32.2%], respectively), renal disease (22477 [22.1%] vs 1977 [9.0%] and 1868 [8.1%], respectively), or history of stroke (16 973 [16.7%] vs 2638 [12.0%] and 2679 [11.6%], respectively) (Table 1). Table 2 shows differences in the prevalence of MCC by anticoagulant type, based on the 3 MCC definitions. For all 3 MCC definitions, warfarin users were consistently more likely to have high MCC compared with the other 2 anticoagulant users.

**Table 1.** Characteristics of Patients Initiating Dabigatran (150 mg), Rivaroxaban (20 mg), or Warfarin Within 90 Days of Initial Diagnosis of Atrial Fibrillation

Characteristic	No. (%)		
	Dabigatran 150 mg (n = 21 979)	Rivaroxaban 20 mg (n = 23 177)	Warfarin (n = 101 715)
Demographics			
Age, mean (SD)	75.83 (6.4)	75.75 (6.4)	78.45 (7.2)
>85 y	2348 (10.7)	2471 (10.7)	22 827 (22.4)
Female	11 239 (51.1)	11 571 (49.9)	58 327 (57.3)
Dual Medicaid enrollment	3740 (17.0)	3204 (13.8)	24 412 (24.0)
Race, %			
White	19 723 (89.7)	21 019 (90.7)	88 544 (87.1)
Black	768 (3.5)	744 (3.2)	5420 (5.3)
Hispanic	824 (3.7)	851 (3.7)	4689 (4.6)
Other	664 (3.0)	563 (2.4)	3062 (3.0)
Comorbid conditions			
Heart failure	5310 (24.2)	5405 (23.3)	37 698 (37.1)
Cardiomyopathy	1468 (6.7)	1673 (7.2)	9752 (9.6)
Other dysrhythmia	7133 (32.5)	7738 (33.4)	36 489 (35.9)
Implantable device	1036 (4.7)	1282 (5.5)	6742 (6.6)
Peripheral vascular disease	4056 (18.5)	4335 (18.7)	25 507 (25.1)
Hypertension	18 399 (83.7)	19 548 (84.3)	87 452 (86.0)
Diabetes	7194 (32.7)	7468 (32.2)	38 040 (37.4)
Renal disease	1977 (9.0)	1868 (8.1)	22 477 (22.1)
Liver disease	861 (3.9)	947 (4.1)	4856 (4.8)
Stroke or TIA	2638 (12.0)	2679 (11.6)	16 973 (16.7)
Previous major bleeding			
Intracranial	101 (0.5)	102 (0.4)	879 (0.9)
Gastrointestinal	5577 (25.4)	5999 (25.9)	29 342 (28.8)
Medications in prior 90 d			
Statin	9842 (44.8)	10 399 (44.9)	44 000 (43.3)
Antiplatelet	1074 (4.9)	1136 (4.9)	6452 (6.3)
PPIs	4366 (19.9)	4686 (20.2)	22 117 (21.7)
NSAIDs	2933 (13.3)	2970 (12.8)	11 667 (11.5)
Prior health services use			
Prior inpatient hospital, mean (SD), d	2.40 (4.9)	2.36 (5.3)	4.95 (8.6)
Skilled nursing facility	846 (3.8)	840 (3.6)	9454 (9.3)
AF diagnosed during inpatient episode	8940 (40.6)	8942 (38.6)	51 054 (50.2)

Abbreviations: AF, atrial fibrillation; NSAIDs, nonsteroidal anti-inflammatory drugs; PPIs, proton pump inhibitors; TIA, transient ischemic attack.

**Table 2.** Multicomorbidity and Illness Burden by Drug

Comorbidity Measure	No. (%)		
	Dabigatran, 150 mg (n = 21 979)	Rivaroxaban, 20 mg (n = 23 177)	Warfarin (n = 101 715)
CHA_2_DS_2_-VASc score, mean (SD)^a^	4.32 (1.65)	4.31 (1.63)	4.93 (1.72)
Low (0-3)	7273 (33.1)	7694 (33.1)	20 846 (20.5)
Moderate (4-5)	9631 (43.8)	9631 (43.8)	44 087 (43.3)
High (≥6)	5075 (23.1)	5230 (22.6)	36 782 (36.2)
HAS-BLED bleeding risk score, mean (SD)^b^	1.64 (0.79)	1.63 (0.78)	1.87 (0.90)
Low (0-1)	11 483 (52.3)	12 254 (52.9)	42 077 (41.4)
Moderate (2)	7520 (34.2)	7829 (33.8)	37 291 (36.7)
High (≥3)	2976 (13.5)	3094 (13.4)	22 347 (22.0)
Gagne multicomorbidity score, mean (SD)^c^	3.02 (2.21)	2.99 (2.21)	4.19 (2.86)
Low (0-2)	11 249 (51.2)	12 022 (51.9)	34 843 (34.3)
Moderate (3-4)	6211 (28.3)	6512 (28.1)	27 524 (27.1)
High (≥5)	4519 (20.6)	4643 (20.0)	39 348 (38.7)

^a^ CHA_2_DS_2_-VASc score ranges from 0 to 9; higher scores indicate higher risk of stroke, transient ischemic attack, or embolism.

^b^ HAS-BLED score ranges from 0 to 9; higher scores indicate higher bleeding risk.

^c^ Gagne multicomorbidity score ranges from 0 to 24; higher scores indicate higher risk of death.

### Unadjusted Outcomes Rates

Prior to propensity matching, there were 320, 321, and 2101 strokes in patients who initiated dabigatran, rivaroxaban, and warfarin, respectively, with rates per 100 patient-years of 1.47, 1.40, and 2.09, respectively (Table 3). Irrespective of the score used to define MCC, stroke rates were lowest in patients with low MCC. The high MCC subgroups had the highest stroke rates in all 3 anticoagulant groups. When the 3 anticoagulants were compared, in general, unadjusted rates of stroke were higher for patients receiving warfarin compared with patients receiving dabigatran or rivaroxaban, regardless of MCC subgroup.

**Table 3.** Number of Outcome Events and Rates per 100 Patient-Years of Follow-up Prior to Matching by Comorbidity Level

Comorbidity Measure	No. (%)		
	Dabigatran, 150 mg (n = 21 979)	Rivaroxaban, 20 mg (n = 23 177)	Warfarin (n = 101 715)
No. of ischemic strokes			
All patients	216 (1.46)	183 (1.39)	1328 (2.09)
CHA_2_DS_2_-VASc score^a^			
Low	34 (0.68)	31 (0.71)	122 (0.88)
Moderate	95 (1.45)	78 (1.33)	448 (1.58)
High	87 (2.67)	74 (2.53)	758 (3.52)
HAS-BLED bleeding risk score^b^			
Low	84 (1.06)	73 (1.03)	379 (1.36)
Moderate	77 (1.53)	63 (1.43)	523 (2.27)
High	55 (2.91)	47 (2.84)	426 (3.35)
Gagne comorbidity score^c^			
Low	91 (1.14)	77 (1.07)	387 (1.60)
Moderate	56 (1.32)	58 (1.57)	390 (2.19)
High	69 (2.64)	48 (2.06)	551 (2.55)
No. of MH			
All patients	502 (3.40)	615 (4.69)	1358 (5.31)
CHA_2_DS_2_-VASc score^a^			
Low	80 (1.60)	95 (2.17)	395 (2.88)
Moderate	233 (3.56)	290 (4.96)	1385 (4.92)
High	189 (5.83)	230 (7.94)	1578 (7.38)
HAS-BLED bleeding risk score^b^			
Low	205 (2.60)	242 (3.41)	1012 (3.65)
Moderate	183 (3.65)	228 (5.19)	1267 (5.53)
High	114 (6.09)	145 (8.88)	1079 (8.58)
Gagne comorbidity score^c^			
Low	166 (2.09)	207 (2.89)	738 (3.06)
Moderate	154 (3.65)	190 (5.18)	878 (4.96)
High	182 (7.02)	218 (9.52)	1742 (8.14)
No. of GIH			
All patients	453 (3.07)	533 (4.06)	2597 (4.09)
CHA_2_DS_2_-VASc score^a^			
Low	70 (1.40)	78 (1.78)	286 (2.08)
Moderate	207 (3.16)	256 (4.38)	1075 (3.81)
High	176 (5.42)	199 (6.85)	1236 (5.76)
HAS-BLED bleeding risk score^b^			
Low	187 (2.37)	206 (2.90)	757 (2.72)
Moderate	163 (3.25)	199 (4.53)	1003 (4.36)
High	103 (5.49)	128 (7.82)	837 (6.62)
Gagne comorbidity score^c^			
Low	146 (1.83)	172 (2.40)	528 (2.18)
Moderate	140 (3.32)	169 (4.60)	659 (3.71)
High	167 (6.44)	192 (8.36)	1410 (6.57)
No. of other (nongastrointestinal) major hemorrhage			
All patients	54 (0.36)	85 (0.64)	801 (1.26)
CHA_2_DS_2_-VASc score^a^			
Low	11 (0.22)	17 (0.39)	115 (0.83)
Moderate	28 (0.42)	36 (0.61)	325 (1.14)
High	15 (0.46)	32 (1.09)	361 (1.66)
HAS-BLED bleeding risk score^b^			
Low	19 (0.24)	37 (0.52)	260 (0.93)
Moderate	22 (0.44)	30 (0.68)	284 (1.22)
High	13 (0.68)	18 (1.08)	257 (2.01)
Gagne comorbidity score^c^			
Low	20 (0.25)	35 (0.49)	218 (0.90)
Moderate	16 (0.38)	23 (0.62)	225 (1.26)
High	18 (0.68)	27 (1.16)	358 (1.65)
No. of AMI			
All patients	125 (0.84)	108 (0.82)	849 (1.33)
CHA_2_DS_2_-VASc score^a^			
Low	22 (0.44)	19 (0.43)	84 (0.61)
Moderate	46 (0.70)	45 (0.76)	334 (1.18)
High	57 (1.74)	44 (1.50)	431 (1.99)
HAS-BLED bleeding risk score^b^			
Low	47 (0.59)	43 (0.60)	250 (0.89)
Moderate	47 (0.93)	40 (0.90)	316 (1.37)
High	31 (1.63)	25 (1.50)	283 (2.22)
Gagne comorbidity score^c^			
Low	46 (0.58)	40 (0.56)	179 (0.74)
Moderate	35 (0.82)	32 (0.86)	221 (1.24)
High	44 (1.67)	36 (1.54)	449 (2.07)
No. of deaths			
All patients	393 (2.64)	417 (3.15)	4282 (6.67)
CHA_2_DS_2_-VASc score^a^			
Low	64 (1.28)	86 (1.96)	506 (3.65)
Moderate	180 (2.73)	190 (3.22)	1603 (5.62)
High	149 (4.53)	141 (4.78)	2173 (9.96)
HAS-BLED bleeding risk score^b^			
Low	159 (2.00)	178 (2.49)	1304 (4.65)
Moderate	166 (3.29)	157 (3.54)	1673 (7.19)
High	68 (3.57)	82 (4.91)	1305 (10.15)
Gagne comorbidity score^c^			
Low	92 (1.15)	88 (1.22)	511 (2.10)
Moderate	104 (2.44)	112 (3.02)	824 (4.59)
High	197 (7.48)	217 (9.28)	2947 (13.50)

Abbreviations: AMI, acute myocardial infarction; GIH, gastrointestinal hemorrhage; MH, major hemorrhage.

^a^ CHA_2_DS_2_-VASc score ranges from 0 to 9; higher scores indicate higher risk of stroke, transient ischemic attack, or embolism. Category ranges are low (0-3), moderate (4-5), and high (≥6).

^b^ HAS-BLED score ranges from 0 to 9; higher scores indicate higher bleeding risk. Category ranges are low (0-1), moderate (2), or high (≥3).

^c^ Gagne multicomorbidity score ranges from 0 to 24; higher scores indicate higher risk of death. Category ranges are low (0-2), moderate (3-4), or high (≥5).

Overall unadjusted rates of major hemorrhage (MH) were 3.42, 4.74, and 5.45 per 100 patient-years of follow-up in patients receiving dabigatran, rivaroxaban, or warfarin, respectively. Rates of MH were highest in patients with high MCC, regardless of which measure was used to define MCC. In contrast to stroke, rates of bleeding were not consistently higher among warfarin users, compared with rivaroxaban and dabigatran users. Among patients with high MCC, rivaroxaban users had the highest rates of MH. In contrast, dabigatran users had the lowest rates of major bleeding, regardless of MCC level. Gastrointestinal hemorrhage accounted for the majority of bleeding events, with rates of 3.09, 4.12, and 4.13 per 100 patient-years in patients receiving dabigatran, rivaroxaban, or warfarin, respectively. Among patients with moderate or high MCC, GIH rates were consistently higher in patients receiving rivaroxaban compared with patients receiving either dabigatran or warfarin. There was little difference in GIH rates among patients with low MCC. Warfarin users experienced higher rates of MI and death in all MCC subgroups regardless of the definition used.

### Risk-Adjusted Outcome Rates

We determined that an unmeasured confounder that had 0.5 greater prevalence among patients receiving rivaroxaban compared with warfarin (ie, 50 percentage points more likely in rivaroxaban), and increased the risk of GIH by 3.7 per 100 patient-years, or had 0.2 greater prevalence among patients receiving rivaroxaban compared with warfarin, and increased the risk of GIH by 9.3 per 100 patient-years, would explain the observed difference in GIH rates among high-risk patients receiving rivaroxaban or warfarin.

Table 4 shows the relative hazard of each outcome in propensity-matched samples. There were no significant differences in ischemic stroke between matched patients receiving dabigatran, rivaroxaban, or warfarin in all MCC levels, regardless of MCC definition in this Medicare population. In patients with low MCC, MH was less likely among dabigatran users, relative to those who initiated warfarin. For example, among patients with high CHA_2_DS_2_-VASc scores receiving dabigatran and warfarin, 28% of all GIH events occurred within 30 days of initiating anticoagulation, while 36% of rivaroxaban GIH events occurred within 30 days of initiating the drug. Patients receiving dabigatran also had significantly lower bleeding rates compared with patients receiving rivaroxaban, particularly among patients with moderate and high MCC levels. Patients receiving rivaroxaban had higher rates of GIH compared with patients receiving warfarin, particularly with moderate and high MCC. Rates of other MH were generally lower among dabigatran users compared with warfarin users, irrespective of MCC level. There were no differences in acute MI events by drug. Finally, the relative hazards of death were generally lower for patients receiving dabigatran relative to warfarin or for patients receiving rivaroxaban relative to warfarin, and did not differ for patients receiving dabigatran relative to rivaroxaban (Figure).

**Table 4.** Relative Hazard of Each Outcome in Propensity-Matched Samples by Comorbidity Level

Comorbidity Measure	Dabigatran, 150 mg vs Warfarin		Rivaroxaban, 20 mg vs Warfarin		Rivaroxaban, 20 mg vs Dabigatran, 150 mg	
	HR (95% CI)	*P* Value	HR (95% CI)	*P* Value	HR (95% CI)	*P* Value
Relative hazard of ischemic stroke						
HAS-BLED bleeding risk score^a^						
Low	1.16 (0.85-1.60)	.35	1.03 (0.74-1.44)	.85	0.89 (0.64-1.22)	.46
Moderate	0.76 (0.56-1.03)	.08	0.69 (0.50-0.95)	.03	0.91 (0.65-1.27)	.58
High	1.26 (0.84-1.89)	.26	1.15 (0.76-1.75)	.51	0.91 (0.61-1.35)	.65
CHA_2_DS_2_-VASc stroke risk score^b^						
Low	0.93 (0.59-1.49)	.78	0.87 (0.54-1.41)	.57	0.93 (0.57-1.52)	.77
Moderate	1.21 (0.90-1.64)	.20	1.06 (0.77-1.45)	.73	0.87 (0.64-1.18)	.38
High	0.87 (0.65-1.16)	.33	0.81 (0.59-1.10)	.17	0.93 (0.68-1.27)	.65
Gagne comorbidity score^c^						
Low	0.87 (0.66-1.16)	.34	0.78 (0.58-1.05)	.11	0.89 (0.66-1.22)	.48
Moderate	0.75 (0.52-1.07)	.11	0.84 (0.59-1.19)	.33	1.12 (0.77-1.63)	.55
High	0.99 (0.70-1.38)	.93	0.74 (0.51-1.07)	.11	0.75 (0.52-1.09)	.13
Relative hazard of any MH						
HAS-BLED bleeding risk score^a^						
Low	0.83 (0.69-1.00)	.05	0.98 (0.81-1.17)	.79	1.18 (0.97-1.43)	.09
Moderate	0.91 (0.74-1.11)	.36	1.20 (0.98-1.45)	.07	1.32 (1.08-1.61)	.01
High	0.83 (0.64-1.07)	.15	1.09 (0.85-1.39)	.49	1.31 (1.02-1.69)	.03
CHA_2_DS_2_-VASc stroke risk score^b^						
Low	0.62 (0.47-0.83)	<.001	0.81 (0.61-1.06)	.13	1.30 (0.95-1.76)	.10
Moderate	0.91 (0.77-1.09)	.33	1.13 (0.95-1.34)	.16	1.24 (1.04-1.48)	.02
High	0.91 (0.74-1.11)	.34	1.16 (0.96-1.41)	.13	1.28 (1.05-1.56)	.01
Gagne comorbidity score^c^						
Low	0.85 (0.69-1.05)	.13	1.12 (0.92-1.37)	.25	1.31 (1.07-1.62)	.01
Moderate	0.85 (0.68-1.06)	.15	1.09 (0.88-1.34)	.42	1.28 (1.03-1.59)	.03
High	0.93 (0.76-1.14)	.50	1.15 (0.95-1.40)	.16	1.24 (1.01-1.51)	.04
Relative hazard of GIH						
HAS-BLED bleeding risk score^a^						
Low	1.10 (0.89-1.35)	.39	1.21 (0.98-1.49)	.08	1.10 (0.90-1.35)	.36
Moderate	0.98 (0.79-1.22)	.87	1.26 (1.02-1.56)	.03	1.28 (1.04-1.59)	.02
High	1.07 (0.80-1.42)	.65	1.36 (1.04-1.79)	.03	1.28 (0.98-1.67)	.08
CHA_2_DS_2_-VASc stroke risk score^b^						
Low	0.71 (0.52-0.97)	.03	0.84 (0.61-1.14)	.27	1.18 (0.84-1.65)	.33
Moderate	1.06 (0.87-1.29)	.55	1.30 (1.08-1.58)	.01	1.23 (1.01-1.48)	.04
High	1.08 (0.87-1.35)	.46	1.28 (1.04-1.59)	.02	1.18 (0.96-1.46)	.11
Gagne comorbidity score^c^						
Low	1.07 (0.84-1.35)	.59	1.30 (1.03-1.63)	.03	1.22 (0.97-1.52)	.09
Moderate	1.01 (0.79-1.28)	.96	1.25 (1.00-1.58)	.05	1.25 (0.99-1.57)	.06
High	1.11 (0.89-1.39)	.36	1.28 (1.03-1.60)	.03	1.16 (0.94-1.43)	.18
Relative hazard of other major (non-GI hemorrhage)						
HAS-BLED bleeding risk score^a^						
Low	0.24 (0.15-0.40)	<.001	0.47 (0.31-0.71)	<.001	1.92 (1.09-3.39)	.02
Moderate	0.59 (0.34-1.00)	.05	0.86 (0.52-1.41)	.54	1.46 (0.83-2.57)	.19
High	0.31 (0.16-0.57)	<.001	0.46 (0.26-0.81)	.01	1.49 (0.72-3.08)	.28
CHA_2_DS_2_-VASc stroke risk score^b^						
Low	0.36 (0.18-0.72)	<.001	0.65 (0.36-1.18)	.16	1.81 (0.84-3.90)	.13
Moderate	0.45 (0.29-0.70)	<.001	0.58 (0.38-0.89)	.01	1.30 (0.79-2.16)	.31
High	0.34 (0.19-0.61)	<.001	0.80 (0.51-1.27)	.34	2.38 (1.29-4.41)	.01
Gagne comorbidity score^c^						
Low	0.34 (0.20-0.57)	<.001	0.68 (0.45-1.05)	.08	2.02 (1.16-3.51)	.01
Moderate	0.38 (0.21-0.67)	<.001	0.56 (0.33-0.94)	.03	1.49 (0.78-2.84)	.23
High	0.39 (0.22-0.67)	<.001	0.63 (0.39-1.02)	.06	1.64 (0.90-2.98)	.11
Relative hazard of death						
HAS-BLED bleeding risk score^a^						
Low	0.63 (0.51-0.77)	<.001	0.68 (0.56-0.82)	<.001	1.08 (0.87-1.34)	.50
Moderate	0.75 (0.61-0.92)	.01	0.72 (0.58-0.88)	<.001	0.95 (0.76-1.19)	.67
High	0.70 (0.51-0.95)	.02	0.80 (0.60-1.09)	.16	1.15 (0.83-1.60)	.40
CHA_2_DS_2_-VASc stroke risk score^b^						
Low	0.52 (0.39-0.71)	<.001	0.62 (0.47-0.82)	<.001	1.18 (0.85-1.65)	.32
Moderate	0.83 (0.68-1.01)	.06	0.81 (0.66-0.98)	.03	0.98 (0.79-1.20)	.81
High	0.77 (0.62-0.96)	.02	0.70 (0.56-0.87)	<.001	0.91 (0.72-1.15)	.44
Gagne comorbidity score^c^						
Low	0.71 (0.54-0.92)	.01	0.65 (0.49-0.85)	<.001	0.92 (0.68-1.23)	.58
Moderate	0.79 (0.61-1.03)	.08	0.84 (0.65-1.08)	.18	1.06 (0.81-1.39)	.68
High	0.71 (0.59-0.86)	<.001	0.74 (0.62-0.89)	<.001	1.04 (0.85-1.27)	.69

Abbreviations: GIH, gastrointestinal hemorrhage; HR, hazard ratio; MH, major hemorrhage.

^a^ CHA_2_DS_2_-VASc score ranges from 0 to 9; higher scores indicate higher risk of stroke, transient ischemic attack, or embolism.

^b^ HAS-BLED score ranges from 0 to 9; higher scores indicate higher bleeding risk.

^c^ Gagne multicomorbidity score ranges from 0 to 24; higher scores indicate higher risk of death.

**Figure.**
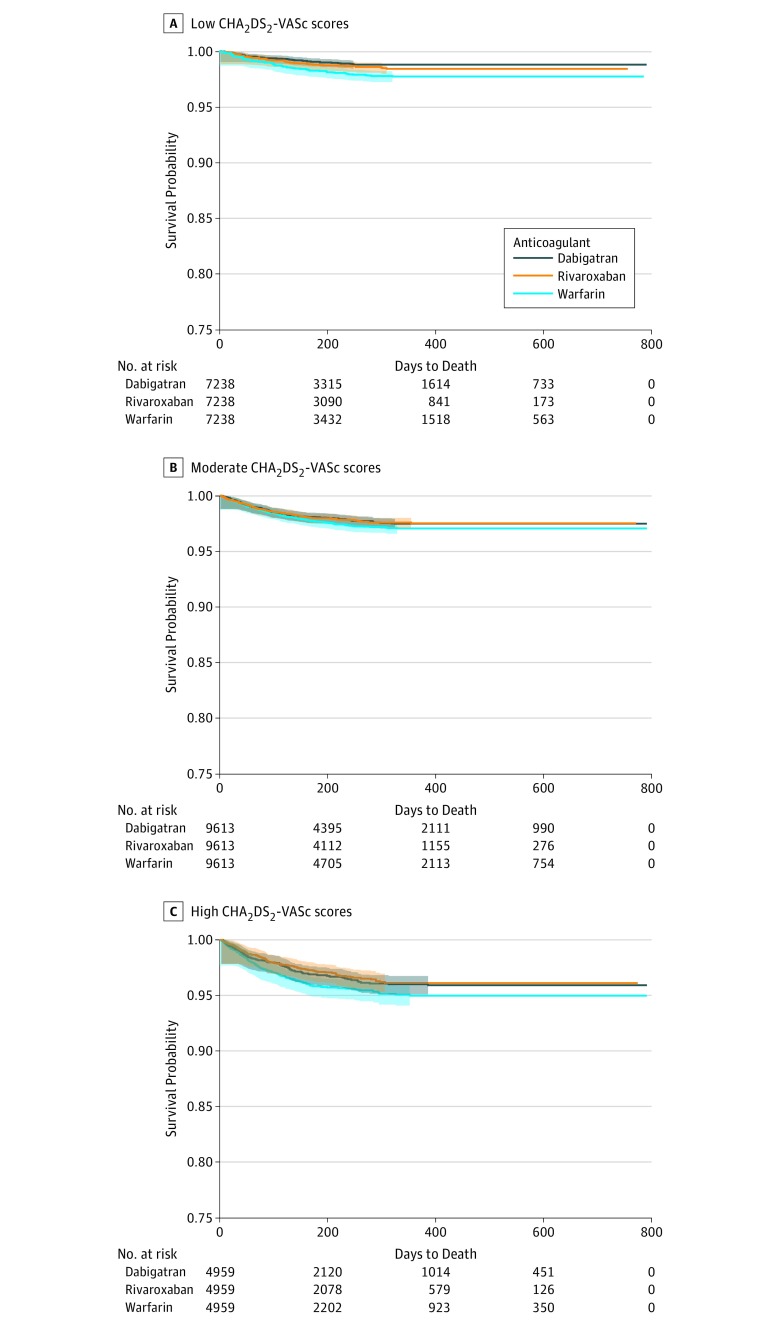
Survival Kaplan-Meier Curves for Death by Anticoagulant Type in Patients With Low, Medium, and High CHA_2_DS_2_-VASc Scores CHA_2_DS_2_-VASc score ranges from 0 to 9; higher scores indicate higher risk of stroke, transient ischemic attack, or embolism. Category ranges are low (0-3) (A), moderate (4-5) (B), and high (≥6) (C).

## Discussion

This study assessed the comparative effectiveness of OAC in a large cohort of elderly Medicare beneficiaries with AF classified as having a low, moderate, and high burden of MCC. We report several important findings. First, there were no significant differences in efficacy of warfarin, dabigatran, and rivaroxaban in adjusted stroke and MI rates across MCC subgroups in this Medicare population. Second, adjusted rates of any MH were significantly lower in dabigatran users compared with rivaroxaban users in patients with moderate to high burden of MCC. Third, rates of GIH were higher in rivaroxaban users compared with the other 2 drugs, mainly in patients with moderate to high MCC. On the other hand, rates of non-GIH were lowest in dabigatran users compared with the other 2 drugs across most MCC levels. Last, mortality rates were generally lower in both rivaroxaban and dabigatran users compared with warfarin users in all MCC subgroups.

With advancing medical treatments, elderly patients live longer with increasingly complex medical conditions and coexisting comorbidities.^26,27^ Dabigatran and rivaroxaban were approved for stroke prophylaxis in patients with AF after 2 RCTs.^5,7^ However, RCTs often exclude sick elderly patients with MCC, which limits the external validity and generalizability to the general population.^28,29^ In our study, over 75% of patients with AF had a CHA_2_DS_2_VASc score higher than 4 and more than 32% had a score higher than 6. In contrast, the ROCKET AF trial included 15% of patients with a score higher than 5 and the RE-LY trial included 33% of patients with a score higher than 3. This underscores the importance of the data presented in our study reporting the comparative effectiveness of anticoagulants in real-world populations with MCC.

Several studies have shown that patients with AF have significantly higher prevalence of comorbidities compared with age- and sex- matched controls, and the presence of multiple comorbidities is associated with worse survival.^30,31,32^ In a recent study of more than 3000 patients with AF, the presence of 4 or more comorbidities was associated with 6-fold mortality risk compared with patients with AF with no comorbidities.^33^ In that study, osteoporosis and chronic obstructive pulmonary disease increased mortality significantly.^33^ In another study, neoplasm, renal disease, and chronic obstructive pulmonary disease were the highest contributing factors to mortality.^30^ Other comorbidities associated with worse outcomes include cognitive impairment and frailty. Cognitive impairment may significantly reduce time in therapeutic range (TTR) in patients with AF treated with warfarin,^34^ and may affect compliance with DOACs as well. Frailty has been shown to be related to underprescribing of recommended anticoagulation.^35^ Indeed, the choice of anticoagulant in patients with multiple conditions is complex. Recent data suggest that physicians are more likely to prescribe DOACs to healthier patients with fewer comorbidities,^12^ while higher CHA_2_DS_2_-VASc and HAS-BLED scores are associated with lower probability of DOAC prescription compared with warfarin.^13^ Our findings of similar effectiveness of all 3 anticoagulants for stroke prevention are assuring and will lend confidence to clinicians regarding DOAC use even in complex patients.

Prior findings regarding relative risk of bleeding with DOACs have been mixed. Initial data from the RE-LY trial suggested higher risk of bleeding with dabigatran compared with warfarin in patients 75 years and older.^5^ However, in subsequent analysis, it was shown that risk of intracranial bleeding is actually lower with dabigatran compared with warfarin in this population while extracranial bleeding was similar.^36^ In 2 network meta-analyses which compared rivaroxaban with dabigatran, both agents were associated with similar bleeding rates,^37,38^ while a third meta-analysis found that DOACs are associated with higher risk of GIH compared with warfarin.^39^ These meta-analyses pooled data from clinical trials in contrast to our study, which included real-world patients, including patients with high comorbidity burden. Our study found no difference between GIH rates in warfarin and dabigatran groups although all of our patients were receiving the higher dose of dabigatran. On the other hand, rivaroxaban was associated with higher GIH rates compared with warfarin and dabigatran. This is consistent with previously published data by Graham et al,^40^ where use of rivaroxaban in Medicare patients was associated with higher bleeding complications compared with dabigatran.^40^ Similarly, a recent network meta-analysis of 11 population-based studies also found that rivaroxaban is associated with higher bleeding risk compared with dabigatran but similar bleeding risk to warfarin.^41^ Our report further explains this data by showing this increased risk is likely driven by moderate and high MCC populations.

To understand our results more fully, we further explored details of GIH bleeding in patients with MCC and determined that patients receiving rivaroxaban are somewhat more likely to experience GIH early, compared with patients receiving dabigatran or warfarin. Among patients with high CHA_2_DS_2_-VASc scores receiving dabigatran and warfarin, 28% of all GIH events occurred within 30 days of initiating anticoagulation, while 36% of rivaroxaban GIH events occurred within 30 days of initiating the drug. Moreover, the greatest differential in bleeding risk occurs within 30 days of initiating rivaroxaban; the hazard of bleeding with rivaroxaban was 1.54-fold greater compared with warfarin within 30 days of initiating the drug (*P* = .03), with no difference in GIH rates after 30 days. There was no difference in GIH between dabigatran and either rivaroxaban or warfarin in high-risk patients, regardless of how many days passed since drug initiation.

The finding in our study that rivaroxaban and dabigatran are associated with lower mortality compared with warfarin in high-risk patients is important. In a prior study by our group, we showed that both dabigatran and rivaroxaban were associated with lower mortality compared with warfarin in both sexes.^42^ In this study we further determine that this finding held true in a range of patients with low to high comorbidity burden, adding support to the efficacy of DOACs in high-risk patients. Our study is consistent with other studies demonstrating equivalent efficacy of DOACs in patients with greater comorbidity levels.^39,43^ The application of 3 unique MCC scores that have been designed and externally validated to address embolic (CHA_2_DS_2_-VASc), bleeding (HAS-BLED), and overall (Gagne) mortality risk strengthens our findings. The Gagne score is particularly unique as it was designed to determine short- and long-term mortality in a Medicare population with superior discrimination compared with other commonly used comorbidity scores for general populations.^22^

### Limitations

The strengths of our study include the large sample, the fact that these patients are from a real-world setting for elderly patients in the United States, and the use of propensity matching to address possible confounding variables. However, there are some limitations that need to be outlined. First, there is always the possibility of residual confounding from unmeasured factors in analysis of observational data even after propensity matching. To address this limitation, we used the approach of Vanderweele and Arah^44^ to assess the likelihood that an unmeasured confounder explains our results. For example, in the matched cohort of patients with a high CHA_2_DS_2_-VASc score, GIH bleeding was higher with rivaroxaban compared with warfarin. We determined that an unmeasured confounder that had 0.5 greater prevalence among patients receiving rivaroxaban compared with warfarin (ie, 50 percentage points more likely in rivaroxaban), and increased the risk of GIH by 3.7 per 100 patient-years, or had 0.2 greater prevalence among patients receiving rivaroxaban compared with warfarin, and increased the risk of GIH by 9.3 per 100 patient-years, would explain the observed difference in GIH rates among high-risk patients receiving rivaroxaban or warfarin. We believe it is unlikely that an unmeasured confounder of this magnitude exists. Second, all of our patients were 65 years and older, which might limit generalizability to younger patients. Third, we defined new AF diagnoses by confirming that patients had no previous AF diagnoses or anticoagulant use during the year prior to the initial AF diagnosis date. However, we cannot verify whether these are truly new AF diagnoses, as patients may have been treated for AF more than 12 months prior. Fourth, we lacked granular details such as type of AF (paroxysmal vs permanent), INR levels, and TTR in warfarin users. International normalized ratio levels are important factors for assessing warfarin efficacy, as compliance with INR testing and consistency of TTR affect outcomes for patients receiving warfarin. In real-world experience, not all patients are compliant with regular INR testing or have a consistent TTR. While our results reflect real-world experience, we cannot infer how results might differ in ideal circumstances with consistent TTR and full compliance with INR testing.

## Conclusions

Rivaroxaban, dabigatran, and warfarin are similarly effective for stroke prevention in patients with AF with MCC. Dabigatran use is associated with a lower bleeding risk whereas rivaroxaban use is associated with a higher bleeding risk compared with warfarin use in these high-risk patients with AF. It is reassuring to note the decreased mortality risk associated with DOACs use in these high-risk patients compared with warfarin use which may motivate clinicians to prescribe DOACs in patients with complex illness.

## Notes

Supplement. **eTable.** Variables Used in Propensity Matching
