# Supplementary material for: Assessment of Outcomes of Treatment With Oral Anticoagulants in Patients With Atrial Fibrillation and Multiple Chronic Conditions: A Comparative Effectiveness Analysis
Source: JAMA Netw Open. 2018 Sep 28;1(5):e182870. doi: 10.1001/jamanetworkopen.2018.2870 (PMC6324495; doi:10.1001/jamanetworkopen.2018.2870)
Supplement: Supplement. — eTable. Variables Used in Propensity Matching [file jamanetwopen-1-e182870-s001.pdf]

Supplementary Online Content

Mentias A, Shantha G, Chaudhury P, Vaughan Sarrazin MS. Assessment of outcomes of treatment with oral anticoagulants in patients with atrial fibrillation and multiple chronic conditions: a comparative effectiveness analysis. *JAMA Netw Open*. 2018;1(5):e182870. doi:10.1001/jamanetworkopen.2018.2870

eTable. Variables Used in Propensity Matching

This supplementary material has been provided by the authors to give readers additional information about their work.

© 2018 Mentias A et al. *JAMA Network Open*.

eTable. Variables Used in Propensity Matching

	**high CHADS2-Vasc**	**high CHADS2-Vasc**		**Medium**	**Medium**		**Low**	**Low**
**variable**	**Pre-matching**	**Post matching**	**variable**	**Pre-matching**	**Post matching**	**variable**	**Pre-matching**	**Post matching**
Obesity	0.38	1.29	Obesity	0.46	3.8	Obesity	1.87	2.24
Hypothyroidism	0.29	0.66	Hypothyroidism	1.78	0.99	Hypothyroidism	1.78	1.18
other dysrhythmia	5.1	0.86	other dysrhythmia	4.19	2.6	other dysrhythmia	3.38	2.54
cardiomyopathy	9.11	1.87	cardiomyopathy	5.36	0.84	cardiomyopathy	1.25	3.46
cerebral infarction	4.3	1.02	cerebral infarction	0.18	2.17	cerebral infarction	0.87	0
GI Hemorrhage	5.49	1.19	GI Hemorrhage	3.5	3.57	GI Hemorrhage	0.53	0.56
Intracranial bleeding	4.26	2.01	Intracranial bleeding	0.75	0.2	Intracranial bleeding	0.08	0.82
Any major bleeding	5.94	1.02	Any major bleeding	3.94	3.97	Any major bleeding	1.07	0.9
prior ischemic stroke	0.65	0.58	prior ischemic stroke	3.27	1.31	prior ischemic stroke	1.13	0
previous cerebrovascular disease	10.2	1.06	previous cerebrovascular disease	8.32	2.15	previous cerebrovascular disease	0.78	0.33
hpertension	1.93	1.42	hpertension	2.86	1.25	hpertension	12.9	2.48

© 2018 Mentias A et al. *JAMA Network Open*.

diabetes	1.09	1.51	diabetes	5.78	1.06	diabetes	0.7	0.13
heart failure	23.47	1.06	heart failure	15.09	3.9	heart failure	5.35	3.71
myocardial infarction	16.16	0.05	myocardial infarction	8.27	1.13	myocardial infarction	2.26	1.5
depression	7.4	1.6	depression	4.29	2.55	depression	2.49	1.82
valve disease	9.91	3.39	valve disease	7.81	1.94	valve disease	1.9	0
COPD	9.31	0.73	COPD	11.73	3.59	COPD	9.07	2.5
peripheral vascular disease	4.75	2.01	peripheral vascular disease	6.11	0.66	peripheral vascular disease	2.05	1.35
neurological disorder	10.7	1.21	neurological disorder	7.31	0.13	neurological disorder	6.27	1.6
renal disease	40.69	0.38	renal disease	31.16	0.39	renal disease	15.9	2.67
liver disease	2.94	0.27	liver disease	3.25	2.02	liver disease	2.02	3.32
dementia	11.55	0.24	dementia	8.13	1	dementia	5.34	0.9
pulmonary circulatatory disorder	17.5	2.82	pulmonary circulatatory disorder	15.86	1.93	pulmonary circulatatory disorder	12.79	1.76
fluid or electrolyte imbalance	25.2	0.84	fluid or electrolyte imbalance	21.14	3.29	fluid or electrolyte imbalance	15.05	0.25
weight loss	14.42	3.84	weight loss	12.13	0.51	weight loss	12.28	0.08
implantable cardiac device	3.54	2.13	implantable cardiac device	6.63	1.47	implantable cardiac device	3.11	1.17

© 2018 Mentias A et al. *JAMA Network Open*.

PGP Inhibitor Use	0.43	1.15	PGP Inhibitor Use	2.27	1.35	PGP Inhibitor Use	8.56	3.65
Clopidogrel/antiplatelet use	1.01	1.16	Clopidogrel/antiplatelet use	4	1.21	Clopidogrel/antiplatelet use	1.26	2.1
Statin Use	9.63	2.91	Statin Use	6.63	1.42	Statin Use	10.94	0.44
NSAID Use	10.08	2.52	NSAID Use	5.95	2.69	NSAID Use	3.06	3.93
Proton Pump Inhibitor Use	0.81	1.15	Proton Pump Inhibitor Use	1.42	0.18	Proton Pump Inhibitor Use	1.74	0.96
Prior SNF Stay	20.96	2.93	Prior SNF Stay	13.85	0.54	Prior SNF Stay	8.66	0.54
CHA2DS2-Vasc Score	15.79	0.49	CHA2DS2-Vasc Score	13.05	4.05	CHA2DS2-Vasc Score	5.25	2.91
HAS-BLED Score	14.91	0.65	HAS-BLED Score	13.12	3.33	HAS-BLED Score	7.56	5.5
Number Prior Inpatient Days	34.36	1.03	Number Prior Inpatient Days	30.59	1.49	Number Prior Inpatient Days	22.02	0.2
Number Medications (ingredients)	0.87	0.63	Number Medications (ingredients)	3.21	0.56	Number Medications (ingredients)	0.78	0.12
GAGNE Comorbidity Score	41.94	0.21	GAGNE Comorbidity Score	33.4	4.72	GAGNE Comorbidity Score	23.27	3.15
Prior Extended Care Stay	21.73	1.99	Prior Extended Care Stay	15.65	0.68	Prior Extended Care Stay	9.51	0.65
Patient Age	30.83	1.62	Patient Age	29.72	4.03	Patient Age	20.09	3.39
Female Sex	3.91	0.4	Female Sex	0.51	0.59	Female Sex	2.91	1.15

© 2018 Mentias A et al. *JAMA Network Open*.

White Race	3.28	3.59	White Race	5.15	3.13	White Race	8.71	3.9
Black Race	7.43	0.49	Black Race	7.06	1.16	Black Race	4.8	3.26
Other non-White race	2.13	4.08	Other non-White race	0.86	2.95	Other non-White race	7.07	2.38

© 2018 Mentias A et al. *JAMA Network Open*.

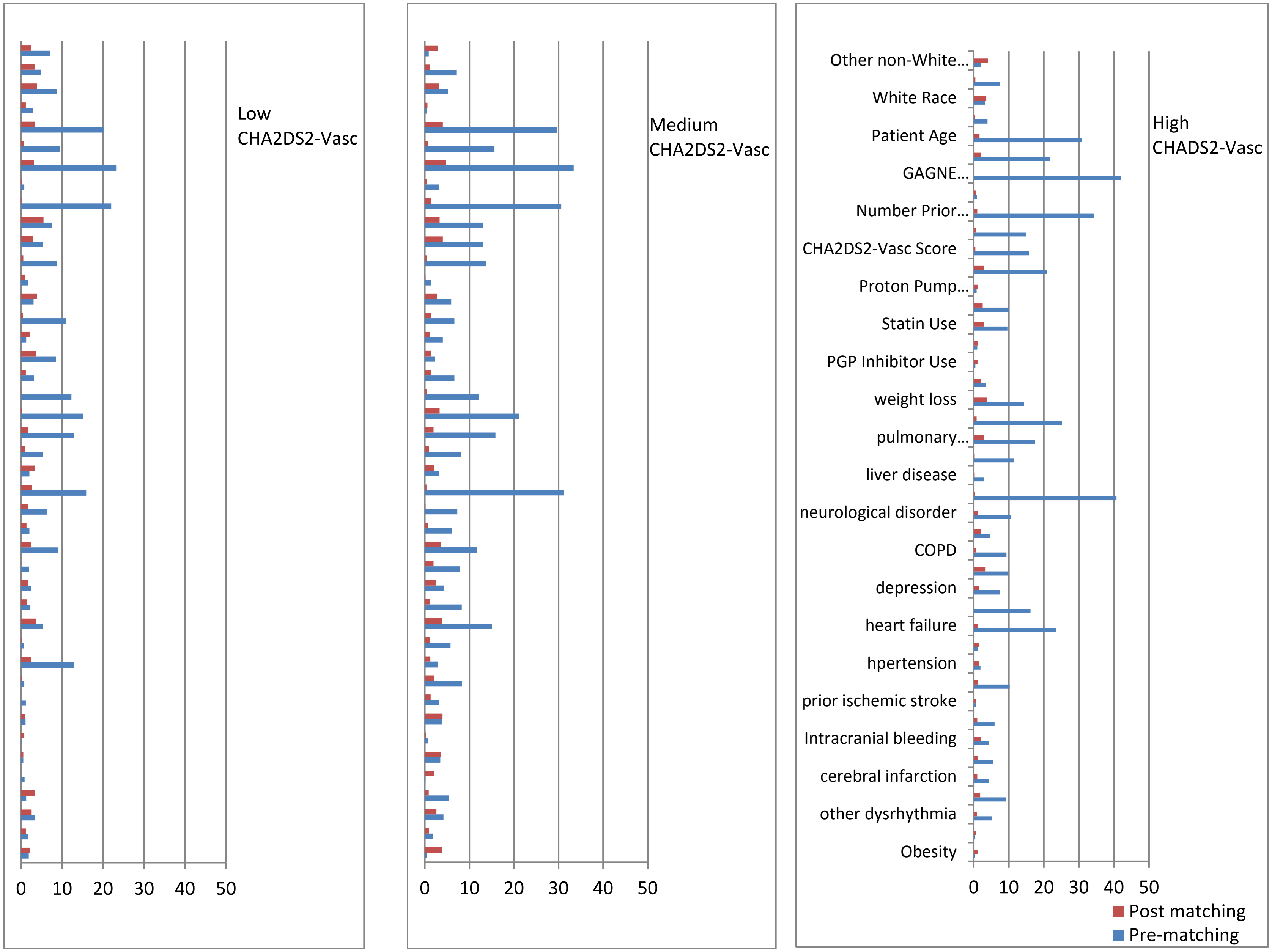

© 2018 Mentias A et al. *JAMA Network Open*.
